# The use of early decision modelling and value of information analysis in an adaptive trial design: results from the OPTIMA preliminary study

**DOI:** 10.1186/1745-6215-16-S2-O19

**Published:** 2015-11-16

**Authors:** Peter Hall, Alison Smith, Claire Hulme, Armando Vargas-Palacios, Janet Dunn, Andrea Marshall, John Bartlett, Rob Stein, David Cameron, Christopher McCabe

**Affiliations:** 1 University of Edinburgh, Edinburgh, UK; 2 University of Leeds, Leeds, UK; 3 University of Warwick, Warwick, UK; 4 University College London, London, UK; 5 Ontario Institute for Cancer Research, Toronto, Canada; 6 University of Alberta, Edmonton, Canada

## Background

The use of decision modelling early in the research and development process for new healthcare technologies may improve research efficiency. Value of information analysis (VOIA) provides a useful tool for assessing the value of conducting further research.

## Objective

To test the feasibility of early modelling within an adaptive randomised controlled trial (RCT), where analysis of preliminary trial data is used to inform a stop-go decision and subsequent trial design.

## Methods

The OPTIMA prelim trial randomised patients with early breast cancer to standard care or test-directed care using Oncotype DX. Additional testing was conducted using five alternative competing multi-parameter tests. A probabilistic decision model was built to assess the cost-effectiveness. VOIA was used to assess the optimal ongoing research strategy to inform an NHS reimbursement decision.

## Results

302 patients were randomised and available for analysis. The cost-effectiveness results suggested multi-parameter tumour testing was likely to be cost-effective. VOIA was able to prioritise tests for inclusion within the ongoing RCT despite the rapid turnaround time required for analysis. The results were highly dependent on modelling assumptions that were unavoidable early in the test development pipeline. Despite difficulties in communicating the unfamiliar concepts underpinning VOIA to the Trial Management Group, it was seen as an informative tool that influenced design decisions.

## Conclusion

Early economic decision modelling and VOIA provides a novel approach to aid the trial design decision making process. It should be considered in future research proposals as a means of improving the return on public research investment within the NHS.

